# Time-resolved proteomic analysis of quorum sensing in *Vibrio harveyi* †
†Electronic supplementary information (ESI) available: Figures and tables. See DOI: 10.1039/c5sc03340c



**DOI:** 10.1039/c5sc03340c

**Published:** 2015-11-23

**Authors:** John D. Bagert, Julia C. van Kessel, Michael J. Sweredoski, Lihui Feng, Sonja Hess, Bonnie L. Bassler, David A. Tirrell

**Affiliations:** a Division of Chemistry and Chemical Engineering , California Institute of Technology , Pasadena , CA 91125 , USA . Email: tirrell@caltech.edu; b Department of Molecular and Cellular Biochemistry , Indiana University , Bloomington , IN 47405 , USA; c Proteome Exploration Laboratory , Beckman Institute , California Institute of Technology , Pasadena , CA 91125 , USA; d Center for Genome Sciences and Systems Biology , Washington University School of Medicine , St. Louis , MO 63108 , USA; e Department of Molecular Biology , Princeton University , Princeton , NJ 08544 , USA; f Howard Hughes Medical Institute , Chevy Chase , MD 20815 , USA

## Abstract

Bacteria use a process of chemical communication called quorum sensing to assess their population density and to change their behavior in response to fluctuations in the cell number and species composition of the community. In this work, we identified the quorum-sensing-regulated proteome in the model organism *Vibrio harveyi* by bio-orthogonal non-canonical amino acid tagging (BONCAT). BONCAT enables measurement of proteome dynamics with temporal resolution on the order of minutes. We deployed BONCAT to characterize the time-dependent transition of *V. harveyi* from individual- to group-behaviors. We identified 176 quorum-sensing-regulated proteins at early, intermediate, and late stages of the transition, and we mapped the temporal changes in quorum-sensing proteins controlled by both transcriptional and post-transcriptional mechanisms. Analysis of the identified proteins revealed 86 known and 90 new quorum-sensing-regulated proteins with diverse functions, including transcription factors, chemotaxis proteins, transport proteins, and proteins involved in iron homeostasis.

## Abstract 2

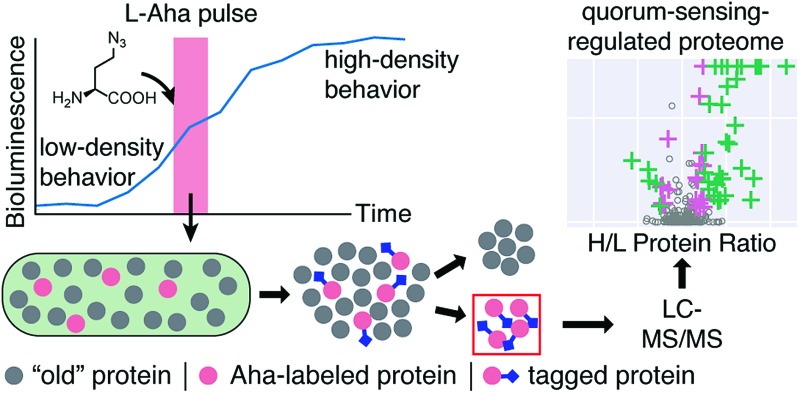
Bio-orthogonal non-canonical amino acid tagging allows time-resolved proteomic analysis of quorum sensing in *Vibrio harveyi*.

## Introduction

Bacteria assess their cell numbers and the species complexity of the community of neighboring cells using a chemical communication process called quorum sensing. Quorum sensing relies on the production, release, accumulation and group-wide detection of signal molecules called autoinducers. Quorum sensing controls genes underpinning collective behaviors including bioluminescence, secretion of virulence factors, and biofilm formation.^1–3^ The model quorum-sensing bacterium *Vibrio harveyi* integrates population-density information encoded in three autoinducers AI-1, CAI-1, and AI-2, which function as intraspecies, intragenus, and interspecies communication signals, respectively.^4–6^ *V. harveyi* detects the three autoinducers using the cognate membrane-bound receptors LuxN, CqsS, and LuxPQ, respectively.^7–9^ At low cell density (LCD), autoinducer concentrations are low, and the unliganded receptors act as kinases, funneling phosphate to the phosphorelay protein LuxU.^10^ LuxU transfers the phosphoryl group to the response regulator protein LuxO, which activates transcription of genes encoding five homologous quorum regulatory small RNAs (*qrr* sRNAs).^11,12^ The Qrr sRNAs post-transcriptionally activate production of the transcription factor AphA and repress production of the transcription factor LuxR. AphA and LuxR are the two master quorum-sensing regulators that promote global changes in gene expression in response to population density changes.^12–15^ At high cell density (HCD), autoinducer binding to the cognate receptors switches the receptors from kinases to phosphatases, removing phosphate from LuxU and, indirectly, from LuxO. Dephosphorylated LuxO is inactive so transcription of the *qrr* sRNA genes ceases. This event results in production of LuxR and repression of AphA.^12^ Thus, the circuitry ensures that AphA is made at LCD, and it controls the regulon required for life as an individual, whereas LuxR is made at HCD, and it directs the program underpinning collective behaviors.

Previous microarray studies examined the transcriptomic response during quorum-sensing transitions. That work showed that AphA and LuxR control over 150 and 600 genes, respectively and ∼70 of these genes are regulated by both transcription factors.^15^ Both AphA and LuxR act as activators and as repressors, and thus the precise pattern of quorum-sensing target gene expression is exquisitely sensitive to fluctuating levels of AphA and LuxR as cells transition between LCD and HCD modes. Developing a comparable understanding of the quorum-sensing-controlled proteome requires measurement of dynamic changes in protein abundance throughout the transition from individual to collective behavior.

In this work, we used the bio-orthogonal non-canonical amino acid tagging (BONCAT) method to track the proteome-wide quorum-sensing response in *V. harveyi* with temporal precision. BONCAT enabled us to identify 176 proteins that are regulated during the transition from individual to collective behavior; 90 of these proteins are in addition to those identified in earlier studies. We show that a broad range of protein functional groups, including those involved in metabolism, transport, and virulence, change during the transition to group behavior. We demonstrate how particular temporal patterns of protein production are linked to particular tiers of the regulatory cascade by comparing the proteomic profiles of the regulon controlled by the post-transcriptional Qrr sRNAs to the regulon controlled by the transcriptional regulator LuxR. Using this approach, we, for example, determined that the *V. harveyi* type VI secretion system is LuxR-regulated.

## Results

**Fig. 1:**
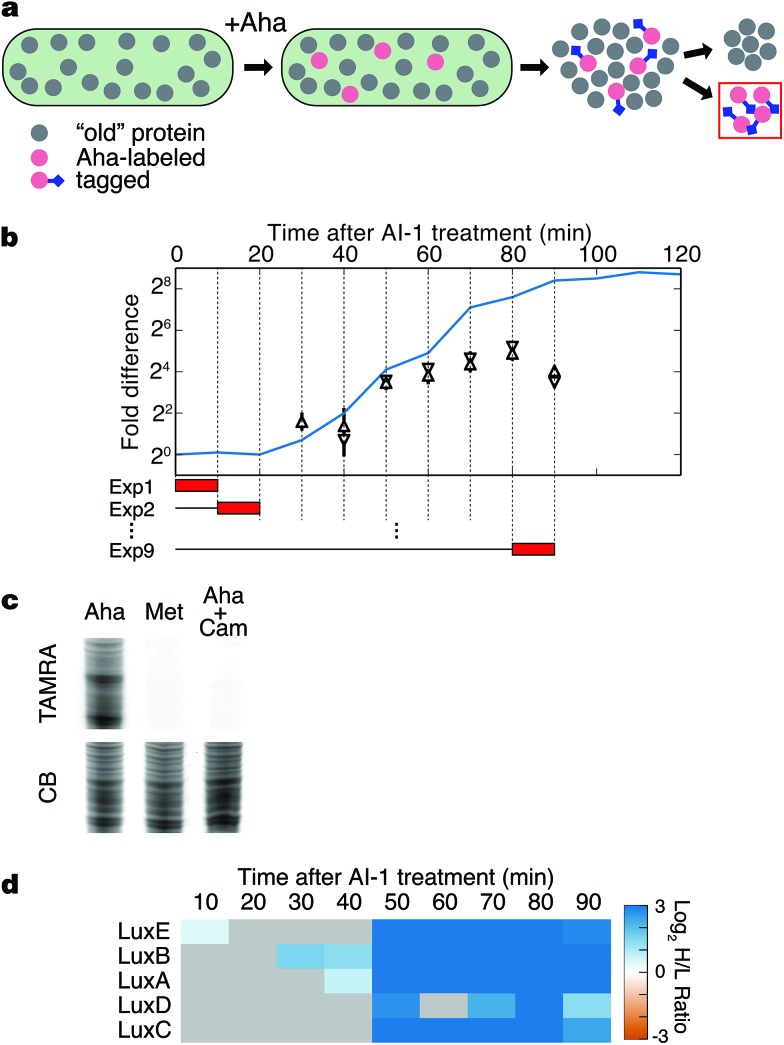
BONCAT analysis of quorum sensing. (a) Treatment of *V. harveyi* TL25 with Aha allows selective tagging and enrichment of newly synthesized proteins. (b) Schematic of BONCAT experiments. The blue line shows bioluminescence emission after AI-1 treatment. Red boxes represent the duration of Aha pulses in separate experiments (Exp 1, Exp 2, *etc.*). BONCAT quantification of the luciferase subunits LuxA (pyramid) and LuxB (reverse pyramid). Error bars denote standard errors of the mean. All proteomic experiments were performed in triplicate. (c) Labeled proteins from a 10 min Aha pulse were conjugated to an alkyne–TAMRA dye (Fig. S1b†) and visualized by in-gel fluorescence. Cultures were treated with Aha, Met, or with Aha together with the protein synthesis inhibitor Cam. CB denotes colloidal blue staining. (d) Heat map showing measured protein production from the *lux* operon. Gray boxes denote samples in which the protein could not be reliably quantified. Chloramphenicol, Cam; TAMRA, tetramethylrhodamine.

The BONCAT method was developed to provide time-resolved analyses of the cellular proteome.^16,17^ In a BONCAT experiment, the non-canonical amino acid l-azidohomoalanine (Aha; Fig. S1a†) is provided to cells and, subsequently, incorporated into proteins in competition with methionine.^18^ Aha-labeled proteins are chemically distinct from the remainder of the protein pool and thus, labeled proteins can be selectively conjugated to affinity tags for enrichment and mass spectrometry analysis (Fig. 1a). Because Aha can be introduced into cells in a well-defined pulse, BONCAT offers excellent temporal resolution and high sensitivity to changes in protein synthesis in response to biological stimuli.^19^

Our goal was to identify time-dependent changes in protein production associated with quorum sensing. We chose to monitor the transition from individual to group behavior in *V. harveyi* because the core transcriptional regulon is well-established, providing a solid foundation for comparisons between transcriptional and translational outputs.^15^ To experimentally manipulate the transition from LCD to HCD, we used *V. harveyi* strain TL25 in which the genes encoding the autoinducer receptors for CAI-1 (*cqsS*) and AI-2 (*luxPQ*) and the AI-1 synthase (*luxM*) have been deleted.^15^ Thus, *V. harveyi* TL25 responds exclusively to exogenously supplied AI-1, which enables precise control over the activation of quorum sensing.

The hallmark phenotypic response controlled by quorum sensing in *V. harveyi* is bioluminescence, which is activated by LuxR during the transition from LCD to HCD.^20^ Thus, we reasoned that light production could serve as a proxy for activation of quorum sensing.^20^ Upon treatment of a culture of *V. harveyi* TL25 with AI-1, bioluminescence increases sharply after 30 min and plateaus at a level 400-fold higher than the pre-addition level after approximately 90 min (Fig. 1b). Detection of Aha incorporation in *V. harveyi* cultures by in-gel fluorescence showed that BONCAT experiments could be performed in this system with a temporal resolution of ten minutes (Fig. 1c). Using the bioluminescence profile as a guide, we combined two techniques, BONCAT and stable isotope labeling with amino acids in cell culture (SILAC), to monitor both increases and decreases in protein synthesis in ten-minute intervals between 0 and 90 min following addition of AI-1 (Fig. 1b and S1c and d†).^19,21^ *V. harveyi* cultures that were not treated with AI-1 served as references for relative quantification. As expected, the production of the luciferase subunits LuxA and LuxB tracked with the bioluminescence profile in cultures treated with AI-1 (Fig. 1b). We detect LuxB at 30 min, slightly before we can detect LuxA. The LuxB measurement is coincident with the first increase in bioluminescence. Between 40 and 50 min, bioluminescence and LuxA and LuxB levels exhibited sharp increases, after which, both continued to climb at slower rates. Between 60 and 90 min, the production rates of LuxA and LuxB remained nearly constant while bioluminescence continued to increase. LuxA and LuxB increased about 8-fold total in response to autoinducer supplementation. This result highlights the fact that BONCAT measures protein synthesis rates during individual time intervals (not total protein abundance), whereas bioluminescence output reports on the total accumulated LuxAB activity.

LuxA and LuxB are encoded by the *lux* operon, which also encodes LuxC, an acyl-CoA reductase, LuxD, an acyl transferase, and LuxE, a long-chain fatty-acid ligase. LuxCDE synthesize the substrate required by the LuxAB luciferase enzyme. All five proteins exhibited large, concurrent increases in translation at 50 min (Fig. 1d). The increase in bioluminescence precedes production of LuxCDE, which suggests some basal level of luciferase substrate is present. The coincidence of the production of LuxA and LuxB with the onset of bioluminescence, and the simultaneous up-regulation of all of the proteins in the *lux* operon validate the BONCAT technique as a reliable method for time-resolved analysis of the quorum-sensing response.

### Detection of quorum-sensing regulators

**Fig. 2:**
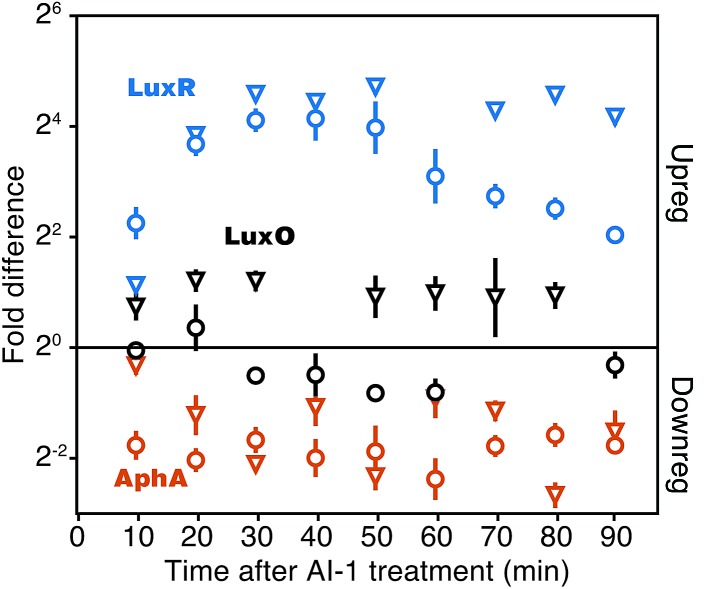
Detection of major quorum-sensing components. Quantitation of LuxR, AphA, and LuxO protein (reverse pyramids) and mRNA (circles). Fold changes are calculated as the difference between cultures treated and not treated with AI-1. LuxR protein quantification was confirmed by manual inspection of MS-MS spectra and calculated peptide retention times (Fig. S2†). Error bars show sample standard errors of the mean.

At the core of the quorum-sensing circuit are the transcriptional regulators LuxO, AphA, and LuxR, which drive quorum-sensing transitions. Expression of *luxO*, *aphA*, and *luxR* are themselves controlled by multiple regulatory feedback loops.^13,15,22–24^ To assess the consequences of addition of AI-1 to *V. harveyi* TL25 on these core regulators, we monitored both mRNA and protein synthesis using qRT-PCR and BONCAT, respectively. LuxO, AphA, and LuxR all showed rapid changes in protein production within 20 min of AI-1 treatment (Fig. 2). AphA and LuxR reached near-maximal differences in translation at the 30 min point; AphA protein production decreased 4-fold and LuxR protein production increased 16-fold. The mRNA levels of *aphA* and *luxR* tracked with those of AphA and LuxR protein changes, with the exception that *luxR* mRNA decreased in abundance between 60 and 90 min while the protein level remained constant. LuxO protein exhibited a consistent 2-fold increase in abundance throughout the time-course, whereas the corresponding mRNA levels slightly decreased. This pattern is consistent with the recent finding that the Qrr sRNAs control *luxO* mRNA through a sequestration mechanism such that the Qrr sRNAs repress LuxO protein production while not significantly altering mRNA abundance.^25^

### Quorum sensing causes global changes in protein synthesis

Using the above protocol for induction of quorum sensing in *V. harveyi* TL25, we next examined the quorum-sensing-controlled proteome using BONCAT to monitor protein synthesis in ten-minute time intervals immediately following addition of AI-1. We collected a total of 700 174 MS/MS spectra and identified 9238 peptides and 1564 unique protein groups (Fig. S3a and b, dataset S1†). Proteins were identified with an average of 6 peptides (median = 4); 88% of proteins were identified by 2 or more peptides (Fig. S3c†). Relative protein abundances at each time point were calculated with an average of 49 unique quantifications (median = 17) (Fig. S3d†). By comparing evidence counts, MS-MS counts, and MS intensities of Met and Aha-containing peptides, we estimated the extent of replacement of Met by Aha to be roughly 15% (Table S1†). Proteins with differences greater than 1.5-fold with false discovery rate-adjusted *p*-values less than 0.05 were considered significant.

**Fig. 3:**
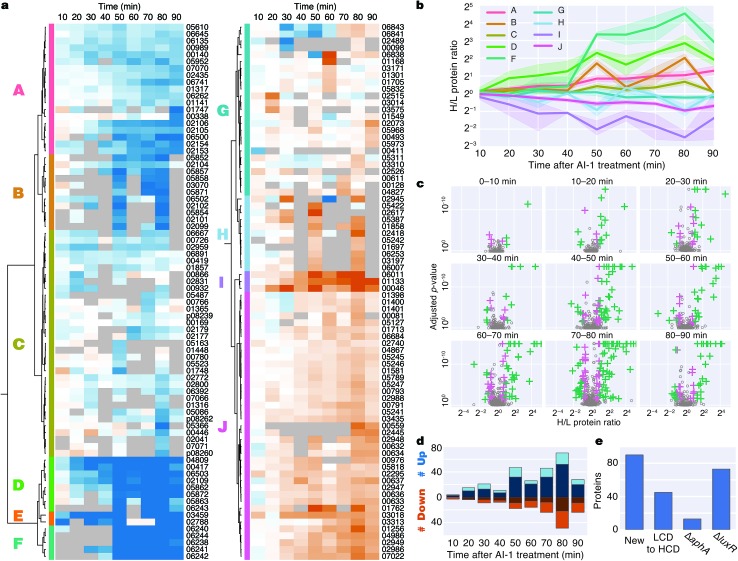
Identification of the quorum-sensing-regulated proteome. (a) Heatmap showing calculated abundances of significantly AI-1-regulated proteins, organized by unsupervised hierarchical clustering. Blue denotes up-regulation and orange denotes down-regulation. Missing ratios are denoted by gray boxes. Proteins with more than 6 missing time points were omitted from the clustering analysis. (b) Temporal behavior of protein clusters. Shaded regions denote 95^th^ percentile confidence intervals. (c) Volcano plots showing outlier proteins for each time point. Proteins with adjusted *p*-values less than 0.05 (Benjamini-Hochberg FDR) are marked by plus signs. Significant proteins with *H*/*L* ratios greater than 2-fold and between 1.5- and 2-fold are designated by green and pink markers, respectively. (d) Total numbers of up-regulated (blue) and down-regulated (orange) proteins at each time point. Dark blue and dark orange portions of bars represent proteins up- and down-regulated more than 2-fold. (e) Identification of new proteins controlled by quorum sensing, and the numbers of outlier proteins identified that belong to the previously established *aphA*, *luxR*, and quorum-sensing (LCD to HCD) regulons.

Induction of quorum sensing altered production of 176 proteins (Fig. 3a). Unsupervised hierarchical clustering partitioned the regulated proteins into 10 groups based on their temporal production profiles (Fig. 3a and b). Proteins from the *lux* operon clustered closely (group F), and LuxR and AphA, which exhibited distinct production profiles, were assigned to very small clusters. Several clusters showed differences in protein production at early time points (groups D, E, I), whereas other clusters changed more abruptly at the 50 min time point (groups B, D, F, H) (Fig. 3b). Differences in protein production between AI-1-treated and control cultures were modest within the first 20 min, with only 7 and 19 significant protein changes at 0–10 min and 10–20 min, respectively. The number of autoinducer-regulated proteins increased with time after induction, with 42–119 proteins altered between 40–90 min after AI-1 treatment (Fig. 3c and d). 90 of the AI-1-regulated proteins are newly associated with quorum sensing in *V. harveyi* (Fig. 3e, Table 2). In total, our analysis identified 278 proteins that are members of the previously established *aphA*, *luxR*, or quorum-sensing regulons.^15^ Interestingly, only 86 of these proteins exhibited significant up- or down-regulation by BONCAT (Fig. S4†).

**Table 1:** Proteins regulated between 0 and 20 min after AI-1 treatment

Gene locus	Peptides	Protein description	Log_2_ *H*/*L* ratio 0–10 min	Log_2_ *H*/*L* ratio 10–20 min
VIBHAR_00411	2	Acetolactate synthase	2.22	ND
VIBHAR_00419	7	Bifunctional protein GlmU	1.08	1.53
VIBHAR_00866	3	Chemotaxis protein	1.17	2.33
VIBHAR_00932	2	DNA polymerase III subunit beta	1.38	2.17
VIBHAR_00989	4	MurNAc-6-P etherase	1.23	1.73
VIBHAR_02109	12	Non-ribosomal peptide synthetase	1.20	2.28
VIBHAR_02295	5	Fumarate/nitrate reduction transcriptional regulator	–1.55	–1.12
VIBHAR_02515	1	Uncharacterized protein	–1.22	–2.80
VIBHAR_02526	3	Uncharacterized protein	–1.51	–1.08
VIBHAR_02788	11	Chemotaxis protein	11.94	ND
VIBHAR_02831	2	Type III secretion protein	1.21	2.04
VIBHAR_03014	4	Superoxide dismutase	–1.04	–1.63
VIBHAR_03256	2	Uncharacterized protein	–1.79	ND
VIBHAR_03575	2	Putative Holliday junction resolvase	–1.03	–2.17
VIBHAR_04809	11	Uncharacterized protein	1.51	3.11
VIBHAR_05607	3	Chitinase	ND	3.37
VIBHAR_06502	17	ATPase	1.36	1.65
VIBHAR_06503	30	Peptidase	1.12	2.07
VIBHAR_06891	3	Ecotin	1.30	1.59

**Table 2:** Proteins newly associated with quorum sensing

Functional association	Proteins
Transcription factor	6
Secretion	8
Metabolism	19
Iron homeostasis	8
Chemotaxis	4
Molecular transport	11
Kinase	4
Protease	3
Other	8
Unknown	19

### Bioinformatic analysis reveals regulation of functionally related protein groups

**Fig. 4:**
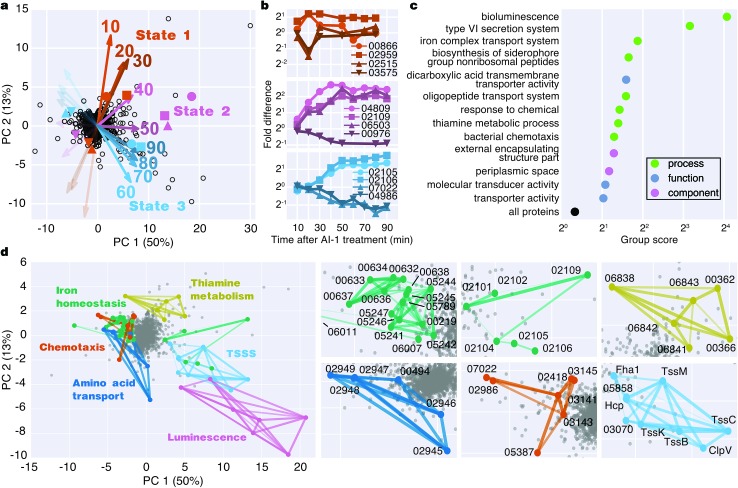
Bioinformatic analysis of the quorum-sensing-regulated proteome. (a) Principal component (PC) analysis of the time-course proteomics data. Percentages show the fractions of the variance for each PC. Vectors show the weights for each time point used to project protein ratios onto the PCs, providing a visual representation of the contribution of each time point to a protein's position in PC space. Weight vectors are positively scaled by a factor of 20 for visibility. Orange, pink, and blue vectors highlight distinct, time-dependent proteomic states after AI-1 treatment. (b) Time-course production profiles of select proteins, represented by colored markers in the PCA biplot, show different timings of protein regulation in response to AI-1 treatment. (c) Gene ontology groups controlled by quorum sensing. Groups were assigned a score based on their member positions on the PCA plot. Significantly AI-1-regulated, non-redundant groups are shown (*p*-value < 0.05). (d) Identification of functionally related, and similarly AI-1-regulated protein groups. A select set of STRING interacting networks were mapped onto the PCA plot with strength and confidence of interactions represented by line thickness and opacity.

To identify major shifts in protein production in response to induction of quorum sensing, we used principal component analysis (PCA) to simplify the dataset by reducing the dimensionality from 9 time points to 2 principal components. Weighting vectors showing the contribution of each time point to the principal components highlighted three distinct proteomic states: (1) an early period in which few proteins changed (10–30 min), (2) a transitional period that included rapid changes in protein production (40–50 min), and (3) a late period in which many proteins exhibited large differences in translation (60–90 min) (Fig. 4a, Table S2†). As confirmation of these states, proteins with principal component coordinates near the 1^st^, 2^nd^, and 3^rd^ sets of vectors exhibited time-course production profiles with punctuated changes at early, middle, and late stages (Fig. 4b). Gene ontology analysis identified 13 protein groups regulated by quorum sensing (Fig. 4c and S5†). Several of these groups were involved in transport, including iron, oligopeptide, and dicarboxylic acid transport. A set of 50 proteins with functional annotations for transporter activity was the largest of enriched ontology groups. Other groups of biological processes included bioluminescence, type VI secretion, siderophore synthesis, thiamine metabolism, and chemotaxis.

To identify groups of functionally related proteins with similar patterns of protein production, we mapped protein interactions from the STRING database onto the PCA plot and scanned for protein networks that localized *via* their principal components (Fig. 4d). Consistent with our gene ontology analysis, we identified interacting protein groups associated with regulation of bioluminescence, type VI secretion, chemotaxis, iron homeostasis, oligopeptide transport, and thiamine metabolism in the quorum-sensing response (Fig. 4d). For example, regarding peptide transport, synthesis of the substrate binding protein of the oligopeptide permease complex, OppA, decreased two-fold between 50–90 min.^26^ Also, a large group of proteins (16) involved in iron transport exhibited decreased production profiles late in the experiment, and a group of iron-regulatory proteins (6) increased in levels. With respect to chemotaxis, we observed both increases and decreases in protein levels: homologs of methyl-accepting chemotaxis proteins and the CheA and CheY signaling proteins decreased, whereas putative methyl-accepting chemotaxis proteins increased in abundance. Taken together, these results suggest an overall quorum-sensing-driven remodeling of iron homeostasis and chemotactic behavior.

### Defining the temporal order of protein regulation in response to quorum sensing

**Fig. 5:**
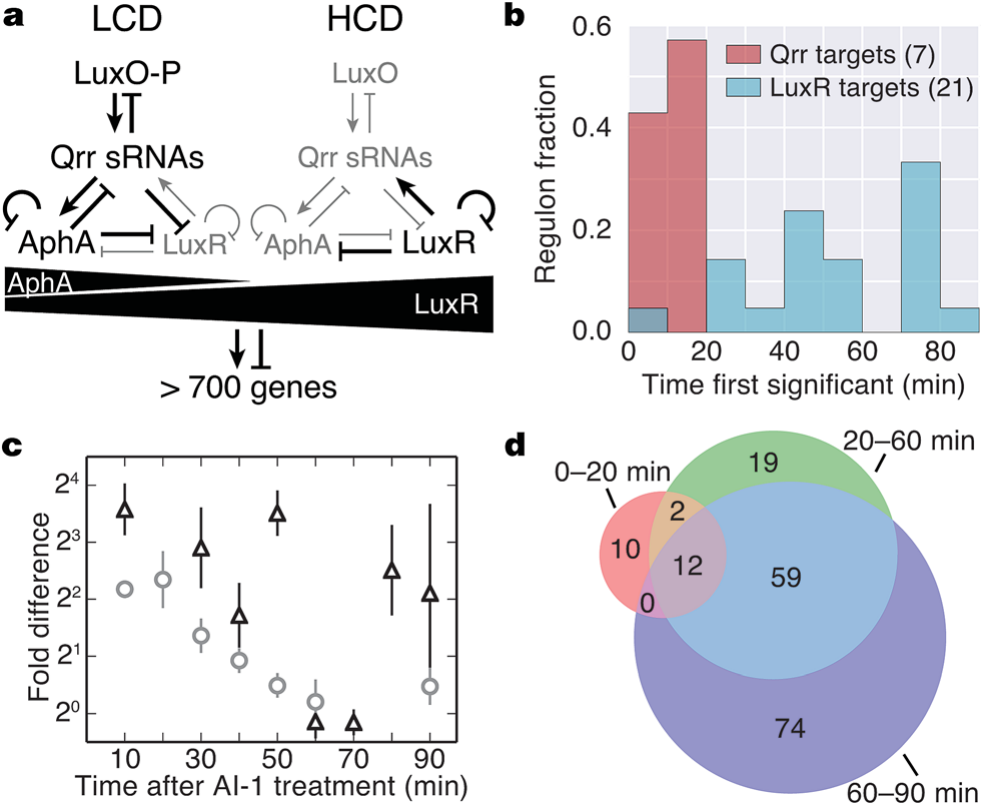
Analysis of the timing of quorum-sensing-regulated protein changes. (a) Diagram of the cytoplasmic portion of the quorum-sensing signal transduction pathway in *V. harveyi*. The horizontal black triangles represent concentration gradients of AphA and LuxR. Panel adapted from van Kessel *et al.* ^15^. (b) Timing of changes of proteins whose genes are direct targets of the Qrr sRNAs or LuxR. Proteins regulated by both the Qrr sRNAs and LuxR were excluded from the list of LuxR targets. (c) Protein (triangles) and mRNA (circles) measurements of VIBHAR_02788 following AI-1 treatment. Error bars designate standard error of the mean. (d) Venn diagram showing the numbers of proteins regulated at early, intermediate, and late times after AI-1 treatment.

The Qrr sRNAs play a central role in dictating the transition between LCD and HCD states by controlling expression of the quorum-sensing transcriptional regulators, AphA, LuxR, and LuxO (Fig. 5a).^22,23^ The Qrr sRNAs directly regulate 16 additional targets outside of the quorum-sensing cascade with functions in virulence, metabolism, polysaccharide export, and chemotaxis.^27^ The direct Qrr targets constitute the set of “first-response” genes and also trigger the later, broader changes in downstream gene expression. With respect to the second wave of quorum-sensing gene expression changes, LuxR plays the major role. Therefore, we compared the temporal patterns of regulation of proteins known to be direct targets of either the Qrr sRNAs or LuxR.^27,28^ We detected regulation of production of seven proteins known to be encoded by Qrr-regulated genes, all of which exhibited significant differences in expression within 20 minutes of AI-1 treatment (Fig. 5b, Table S3†). Conversely, 20 of the 21 LuxR-regulated proteins identified by BONCAT showed differences in production only after at least 30 minutes of AI-1 induction. Thus, the differences in timing between Qrr- and LuxR-regulated genes reflect the underlying structure of the quorum-sensing circuitry. We investigated the protein production profiles of the newly identified proteins to pinpoint additional candidates for regulation by the Qrr sRNAs. We found 19 additional proteins that are regulated within 20 minutes of AI-1 treatment, suggesting that the corresponding mRNAs may be targeted by the Qrr sRNAs (Table 1). The candidates include two putative chemotaxis proteins, the serine protease inhibitor ecotin, the type III secretion protein chaperone SycT, a chitinase, and several other proteins involved in metabolism. Strikingly, the mRNA and protein production of VIBHAR_02788 (a predicted chemotaxis protein) increased 4- and 12-fold, respectively, within the first 10 minutes after AI-1 treatment, suggesting that VIBHAR_02788 is a good candidate for post-transcriptional regulation by the Qrr sRNAs (Fig. 5c).

The mechanisms that control production of quorum-sensing-regulated proteins undoubtedly become more complex as the response progresses. We identified proteins that were regulated at all stages (early (0–20 min), intermediate (20–60 min), and late (60–90 min)) following AI-1 treatment (Fig. 5d, dataset S1†). Differences in the timing of quorum-sensing-regulated proteins suggest that additional regulatory components or mechanisms orchestrate the transition from individual to group behavior. For example, direct LuxR targets were regulated in both the intermediate and late phases, despite the fact that LuxR reaches its peak production at 30 min (Fig. 2a). This result suggests that accumulation of LuxR or additional transcriptional regulators contribute to control of LuxR-regulated genes.

### Quorum sensing regulates type VI secretion proteins in *V. harveyi*

**Fig. 6:**
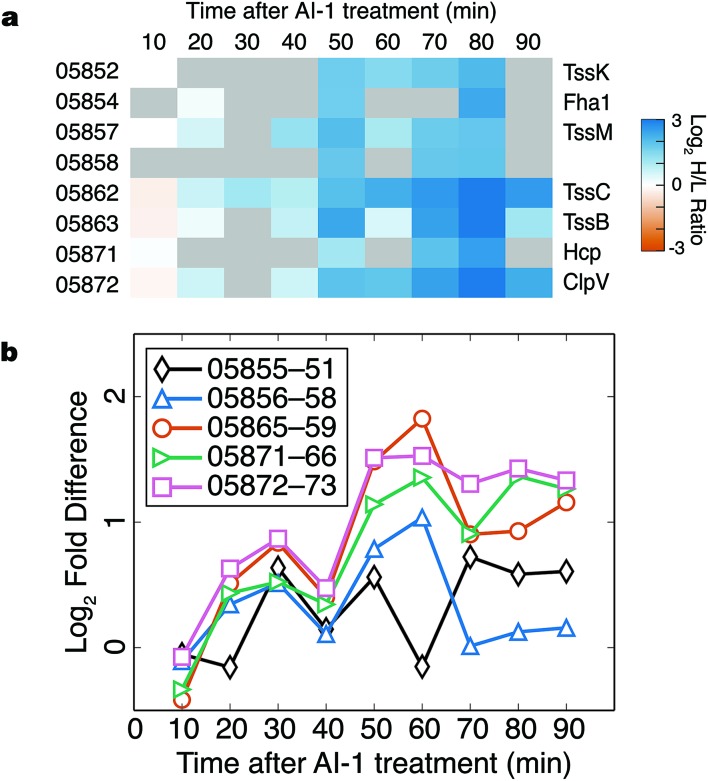
Type VI secretion is controlled by quorum sensing in *V. harveyi*. (a) Heatmap of all quantified TSSS proteins. Values of protein quantification are represented by the colour bar. Gray boxes denote values that could not be reliably quantified. (b) RT-PCR of the five TSSS gene clusters after AI-1 treatment.

Components of the type VI secretion system (TSSS) were among the proteins most strongly up-regulated in response to AI-1 treatment (Fig. 6a). Identified TSSS proteins included the haemolysin co-regulated effector protein (Hcp; VIBHAR_05871), and two additional proteins whose homologs have been implicated in TSSS regulation and Hcp secretion (VIBHAR_05854 and VIBHAR_05858).^29,30^ TSSS proteins exhibited a coordinated increase in production at 50 min, a profile similar to that of LuxCDABE.

In *V. harveyi*, the TSSS homologs are encoded by five putative operons: *VIBHAR_05855–05851*, *VIBHAR_05856–05858*, *VIBHAR_05865–05859*, *VIBHAR_05871–05866*, and *VIBHAR_05872–05873* (Fig. S6a†). Analysis of the mRNA levels of the operons confirmed the increase in expression of TSSS components between 50 to 60 min after AI-induction; timing consistent with second-tier regulation (Fig. 6b). Previous microarray data comparing wild-type, Δ*luxR*, Δ*aphA*, and Δ*luxR* Δ*aphA V. harveyi* strains showed that TSSS gene expression was reduced in Δ*luxR* strains, but expression was not altered in the Δ*aphA* strain, providing evidence that expression of TSSS genes is LuxR-dependent and AphA-independent (Fig. S6b†).^13^ Consistent with this notion, ChIP-seq data identified a LuxR binding site in the bi-directional promoter region of *VIBHAR_05855*–*05856*.^28^ Using electrophoretic mobility shift assays, we confirmed the presence of this LuxR binding site and determined that LuxR binds to two additional promoter regions in the TSSS locus (Fig. S6c†). This result shows that, unlike *Vibrio cholerae* which deploys the Qrr sRNAs to post-transcriptionally regulate TSSS, *V. harveyi* uses LuxR to control TSSS production.^31^ This finding suggests that although both organisms have TSSS under quorum-sensing control, they employ different regulatory strategies to achieve distinct timing of TSSS protein production.

## Discussion and conclusions

Global transcriptomic studies of *V. harveyi* have uncovered a continuum of changes in gene expression during the transition from LCD to HCD. As *V. harveyi* responds to changes in concentrations of autoinducers, shifts in the levels of the regulatory components AphA, LuxR, and the Qrr sRNAs occur, which in turn alter the expression of the downstream genes in the quorum-sensing regulon. Here we used the BONCAT method to measure changes in the quorum-sensing-regulated proteome during the transition from LCD to HCD, with a time-resolution of 10 min. We found correlated changes in production of the LuxCDABE enzymes and in the intensity of bioluminescence produced by the culture, and we observed regulation of the core regulatory components AphA, LuxR, and LuxO. Notably, the increase in LuxO upon induction of quorum sensing occurred at the level of the protein, but not the mRNA, consistent with the hypothesis that the *luxO* mRNA is regulated by sequestration by the Qrr sRNAs.^25^

The time resolution of the BONCAT method allowed us to identify proteins whose rates of synthesis were altered during the early, intermediate, and late stages of the LCD to HCD transition. The proteins found to be regulated within the first 20 min of autoinducer treatment included seven of the 20 known Qrr sRNA targets along with 19 other proteins not previously associated with Qrr regulation. No known Qrr targets were regulated at later times. In contrast, changes in the known LuxR targets occurred between 30 and 90 min following induction. Notably, proteins in the TSSS were up-regulated between 40 and 50 min following autoinducer treatment, suggesting LuxR regulation of type VI secretion in *V. harveyi*; this conclusion was confirmed by electrophoretic mobility shift assays. Several LuxR-regulated genes exhibited changes in protein production only very late in the BONCAT experiment, which suggests either that they are responsive to accumulating LuxR levels, that they are regulated by another transcription factor downstream of LuxR, or that they are co-regulated by other factors.

We found quorum-sensing-dependent changes in 176 proteins that span a broad range of functional groups, including those related to iron homeostasis, molecular transport, metabolism, and chemotaxis. Ninety of these proteins are newly associated with quorum sensing in *V. harveyi*, and expand what is known about the roles that quorum sensing plays in these processes.^13,32^ The remaining 86 proteins are members of the previously established quorum-sensing, AphA, and/or LuxR regulons. Interestingly, nearly 200 other proteins from these regulons were identified by BONCAT but were not significantly up- or down-regulated. For example, the quorum-sensing regulon, which was defined by differences in gene expression between a mutant *V. harveyi* strain locked at LCD and a strain locked at HCD, contains 365 regulated genes as determined by microarray analysis.^15^ We quantified protein expression levels of 127 (35%) of these genes, 45 (35%) of which were significantly regulated. The differences between the genetic and proteomic results may arise, at least in part, from differences in regulation at the levels of mRNA and protein, or from differences in the growth media used in the two experiments (rich (LM) medium in the genetic study *vs.* minimal (AB) medium here).^13,15^ Furthermore, we would not expect the rapid addition of saturating amounts of AI-1 to a *V. harveyi* culture to reproduce precisely the effects of genetically locking the strain into either the LCD or the HCD state. Determining how environmental conditions affect the quorum-sensing response will be important to the development of a full understanding of bacterial communication in complex natural environments.

The BONCAT method has allowed us to identify a diverse set of proteins that respond to the induction of quorum sensing in *V. harveyi*. The method facilitates monitoring of changes in protein synthesis on a time scale of minutes, and enables correlation of those changes with the underlying temporal pattern of regulation of the quorum-sensing response. The approach described here should prove useful in studies of a wide variety of time-dependent cellular processes.

## Experimental

### Cell culture

For each set of experiments, overnight cultures of *V. harveyi* strain TL25 (Δ*luxM* Δ*luxPQ* Δ*cqsS*) was used to inoculate 625 mL of AB minimal medium containing 18 amino acids (–Met, –Lys) at an OD_600_ of 0.003.^15^ The culture was divided into six 100 mL aliquots. Three aliquots were supplemented with “light” Lys and three were supplemented with “heavy” Lys (U–^13^C_6_ U–^15^N_2_ l-lysine, Cambridge Isotope Laboratories). When the aliquoted cultures reached an OD_600_ of 0.1 (∼5 doublings), two “heavy” cultures (replicates 1 and 2) and one “light” culture (replicate 3) were treated with AI-1 at a final concentration of 10 μM (‘AI-1 added’); the other three cultures were left untreated (‘no AI-1 added’). At the specified time intervals, Aha was pulsed into all six cultures at a final concentration of 1 mM. After 10 min of Aha treatment, protein synthesis was halted by the addition of 100 μg mL^–1^ chloramphenicol (Sigma). Cells were pelleted, frozen at –80 °C, and stored for downstream processing. Aha was synthesized as described previously.^33^ Cultures were grown at 30 °C in a shaking incubator at 250 rpm.

### Molecular methods

To measure changes in gene expression following induction of quorum sensing in *V. harveyi* TL25, cultures were grown as described above, divided in half, and AI-1 was added to one of the aliquots. Samples were collected every 10 min and RNA was isolated as described previously.^13^ cDNA synthesis and qRT-PCR were performed as described previously.^22^ The levels of gene expression were normalized to the internal standard *hfq* using either the ΔΔ*C*_T_ method or the standard curve method. At least two replicates were collected for each sample (‘AI-1 added’ or ‘no AI-1 added’). The graphs show the average of those measurements and are calculated as ‘AI-1 added’ divided by ‘no AI-1 added’. Electrophoretic mobility shift assays were performed as previously described.^15^ PCR products were generated using oligonucleotides (Integrated DNA Technologies) listed in Table S4.†

### BONCAT

Cells were lysed by heating in 1% SDS in PBS at 90 °C for 10 min and lysates were cleared by centrifugation. Protein concentrations were determined with the BCA protein quantitation kit (Thermo Scientific), and paired ‘light’ and ‘heavy’ cultures were mixed at equal quantities of total protein. Azide-alkyne click chemistry was performed as described in Hong *et al.* with a 0.1 mM alkyne–DADPS tag and allowed to proceed for 4 h at room temperature (Fig. S1e†).^34^ The DADPS tag was synthesized as described previously.^35^ Proteins were concentrated by acetone precipitation and solubilized in 2% SDS in PBS. Solutions were diluted to 0.15% SDS in PBS, and tagged proteins were captured by incubating with streptavidin UltraLink resin (Thermo Scientific) for 30 min at room temperature. Resin was washed with 35 column volumes of 1% SDS in PBS and 10 column volumes of 0.1% SDS in ddH_2_O. The DADPS tag was cleaved by incubating the resin in 5% formic acid in 0.1% SDS in ddH_2_O for 1 h. Columns were washed with 5 column volumes of 0.1% SDS in H_2_O, during which proteins remained bound, and proteins were subsequently eluted in 15 column volumes of 1% SDS in PBS. Protein enrichment was confirmed by SDS-PAGE, and eluted proteins were concentrated on 3 kDa MWCO spin filters (Amicon).

### In-gel digestion

Concentrated proteins were separated on precast 4–12% polyacrylamide gels (Life Technologies) and visualized with colloidal blue stain (Life Technologies). Lanes were cut into 8 slices and proteins were destained, reduced, alkylated, digested with LysC (Mako), and extracted as described in Bagert *et al.* ^19^ Extracted peptides were desalted with custom-packed C_18_ columns as described in Rappsilber *et al.*, lyophilized, and resuspended in 0.1% formic acid (Sigma).^36^

### Liquid chromatography-mass spectrometric analyses

Liquid chromatography-mass spectrometry and data analyses were carried out on an EASY-nLC-orbitrap mass spectrometer (Thermo Fisher Scientific, Bremen, Germany) as previously described with the following modifications.^37^ For the EASY-nLC II system, solvent A consisted of 97.8% H_2_O, 2% ACN, and 0.2% formic acid and solvent B consisted of 19.8% H_2_O, 80% ACN, and 0.2% formic acid. For the LC-MS/MS experiments, samples were loaded at a flow rate of 500 nL min^–1^ onto a 16 cm analytical HPLC column (75 μm ID) packed in-house with ReproSil-Pur C_18_AQ 3 μm resin (120 Å pore size, Dr Maisch, Ammerbuch, Germany). The column was enclosed in a column heater operating at 30 °C. After *ca.* 20 min of loading time, the peptides were separated with a 60 min gradient at a flow rate of 350 nL min^–1^. The gradient was as follows: 0–30% solvent B (50 min), 30–100% B (1 min), and 100% B (8 min). The orbitrap was operated in data-dependent acquisition mode to alternate automatically between a full scan (*m*/*z* = 300–1700) in the orbitrap and subsequent 10 CID MS/MS scans in the linear ion trap. CID was performed with helium as collision gas at a normalized collision energy of 35% and 30 ms of activation time.

### Protein quantification and ratio statistics

Thermo RAW files were processed with MaxQuant (v. 1.4.1.2) using default parameters and LysC/P as the enzyme. Peptide and protein false discovery rates were fixed at 1% using a target-decoy approach. Additional variable modifications for Met were Aha (–4.9863), l-2,4-diaminobutanoate (–30.9768), a product of Aha reduction, alkyne–DADPS (+835.4300), and 5-hexyn-1-ol (+93.0868), a product of alkyne-DADPS cleavage. Multiplicity was set to 2, and light and heavy (+8.0142) lysine labels were specified for all experiments. Aha and 5-hexyn-1-ol modifications were included in protein quantification. We required protein quantifications to be calculated with at least two evidences for each set of experiments.

Both pooled variances and bootstrap statistical methods were employed as previously described to estimate the individual protein ratio standard errors.^19,38^ First, pooled estimates of peptide variation were calculated separately for peptides with well-characterized ratios and those based on requantification in MaxQuant. Second, standard errors of the overall protein ratios were calculated by generating 1000 bootstrap iterations. These iterations were generated by resampling the replicates and peptides and adding a small amount of random variation to each measurement based on the pooled variance estimates. Once the bootstrapped samples were generated for each protein, the standard error of the protein ratio was calculated from the standard deviation of the bootstrapped iterations. Using the standard error, proteins with ratios significantly different from 1 : 1 were identified using a *Z*-test and *p*-values were adjusted to account for multiple hypothesis testing using the Benjamini and Hochberg method.^39^

### Bioinformatic analysis

Hierarchical clustering was performed with R (v. 3.1.1) using Ward's method.^40^ Confidence intervals (95^th^ percentile) for cluster time-series data were calculated by a bootstrapping approach using the tsplot function from the Python (v. 2.7) module seaborn (v. 0.4.0). Singular value decomposition was computed for PCA with the Python module matplotlib.mlab (v. 1.4.0). Gene ontology analysis was performed using a combination of GO terms and KEGG orthology and module terms. Group scores were defined as the mean of protein distances from the origin of the PCA biplot (PC1 *vs.* PC2). Statistical cutoffs (*p*-value < 0.05) were calculated using a bootstrapping approach that calculates scores for 100 000 groups randomly selected from the total pool of quantified proteins. Cutoffs were calculated individually for each group size (*n* = 4, 5, *etc.*) and groups with fewer than 4 members were excluded. Version 9.1 of the STRING database was used for identifying protein interactions, and interacting networks were identified by manual inspection.^41^

## References

[cit1] Miller M. B., Bassler B. L. (2001). Annu. Rev. Microbiol..

[cit2] Zhu J., Mekalanos J. J. (2003). Dev. Cell.

[cit3] Jayaraman A., Wood T. K. (2008). Annu. Rev. Biomed. Eng..

[cit4] Cao J.-G., Meighen E. A. (1989). J. Biol. Chem..

[cit5] Ng W.-L., Perez L. J., Wei Y., Kraml C., Semmelhack M. F., Bassler B. L. (2011). Mol. Microbiol..

[cit6] Chen X., Schauder S., Potier N., van Dorsselaer A., Pelczer I., Bassler B. L., Hughson F. M. (2002). Nature.

[cit7] Freeman J. A., Lilley B. N., Bassler B. L. (2000). Mol. Microbiol..

[cit8] Henke J. M., Bassler B. L. (2004). J. Bacteriol..

[cit9] Bassler B. L., Wright M., Silverman M. R. (1994). Mol. Microbiol..

[cit10] Freeman J. A., Bassler B. L. (1999). J. Bacteriol..

[cit11] Freeman J. A., Bassler B. L. (1999). Mol. Microbiol..

[cit12] Tu K. C., Bassler B. L. (2007). Genes Dev..

[cit13] Rutherford S. T., van Kessel J. C., Shao Y., Bassler B. L. (2011). Genes Dev..

[cit14] Shao Y., Bassler B. L. (2012). Mol. Microbiol..

[cit15] van Kessel J. C., Rutherford S. T., Shao Y., Utria A. F., Bassler B. L. (2013). J. Bacteriol..

[cit16] Dieterich D. C., Link A. J., Graumann J., Tirrell D. A., Schuman E. M. (2006). Proc. Natl. Acad. Sci. U. S. A..

[cit17] Dieterich D. C., Lee J. J., Link A. J., Graumann J., Tirrell D. A., Schuman E. M. (2007). Nat. Protoc..

[cit18] Kiick K. L., Saxon E., Tirrell D. A., Bertozzi C. R. (2002). Proc. Natl. Acad. Sci. U. S. A..

[cit19] Bagert J. D., Xie Y. J., Sweredoski M. J., Qi Y., Hess S., Schuman E. M., Tirrell D. A. (2014). Mol. Cell. Proteomics.

[cit20] Swartzman E., Silverman M., Meighen E. A. (1992). J. Bacteriol..

[cit21] Ong S.-E., Blagoev B., Kratchmarova I., Kristensen D. B., Steen H., Pandey A., Mann M. (2002). Mol. Cell. Proteomics.

[cit22] Tu K. C., Long T., Svenningsen S. L., Wingreen N. S., Bassler B. L. (2010). Mol. Cell.

[cit23] Teng S.-W., Schaffer J. N., Tu K. C., Mehta P., Lu W., Ong N. P., Bassler B. L., Wingreen N. S. (2011). Mol. Syst. Biol..

[cit24] Tu K. C., Waters C. M., Svenningsen S. L., Bassler B. L. (2008). Mol. Microbiol..

[cit25] Feng L., Rutherford S. T., Papenfort K., Bagert J. D., van Kessel J. C., Tirrell D. A., Wingreen N. S., Bassler B. L. (2015). Cell.

[cit26] He Q., Chen J., Li C. (2011). J. Ocean Univ. China.

[cit27] Shao Y., Feng L., Rutherford S. T., Papenfort K., Bassler B. L. (2013). EMBO J..

[cit28] van Kessel J. C., Ulrich L. E., Zhulin I. B., Bassler B. L. (2013). mBio.

[cit29] Mougous J. D., Gifford C. A., Ramsdell T. L., Mekalanos J. J. (2007). Nat. Cell Biol..

[cit30] Salomon D., Gonzalez H., Updegraff B. L., Orth K. (2013). PLoS One.

[cit31] Shao Y., Bassler B. L. (2014). Mol. Microbiol..

[cit32] Yang Q., Defoirdt T. (2015). Environ. Microbiol..

[cit33] Link A. J., Vink M. K. S., Tirrell D. A. (2007). Nat. Protoc..

[cit34] Hong V., Presolski S. I., Ma C., Finn M. G. (2009). Angew. Chem., Int. Ed..

[cit35] Szychowski J., Mahdavi A., Hodas J. J. L., Bagert J. D., Ngo J. T., Landgraf P., Dieterich D. C., Schuman E. M., Tirrell D. A. (2010). J. Am. Chem. Soc..

[cit36] Rappsilber J., Mann M., Ishihama Y. (2007). Nat. Protoc..

[cit37] Kalli A., Hess S. (2012). Proteomics.

[cit38] Pierce N. W., Lee J. E., Liu X., Sweredoski M. J., Graham R. L. J., Larimore E. A., Rome M., Zheng N., Clurman B. E., Hess S., Shan S., Deshaies R. J. (2013). Cell.

[cit39] Benjamini Y., Hochberg Y. (1995). Journal of The Royal Statistical Society Series B-statistical Methodology.

[cit40] Ward J. H. (1963). J. Am. Stat. Assoc..

[cit41] Jensen L. J., Kuhn M., Stark M., Chaffron S., Creevey C., Muller J., Doerks T., Julien P., Roth A., Simonovic M., Bork P., von Mering C. (2009). Nucleic Acids Res..

