# Supplementary material for: Time-resolved proteomic analysis of quorum sensing in Vibrio harveyi
Source: Chem Sci. 2015 Nov 23;7(3):1797–806. doi: 10.1039/c5sc03340c (PMC4763989; doi:10.1039/c5sc03340c)
Supplement: Supplementary file 1 [file SC-007-C5SC03340C-s001.pdf]

Electronic Supplementary Material (ESI) for Chemical Science.
This journal is © The Royal Society of Chemistry 2015

**Supplementary figures**

Fig. S1. (A) The structure of L-azidohomoalanine (Aha). (B) The structure of TAMRA-alkyne. (C) A combined SILAC-BONCAT approach for quantifying differences in protein translation. Reference cultures were not treated with AI-1 at time 0 min. Otherwise, they were treated identically to experimental cultures. Experiments were performed in triplicate with one isotope label swap experiment. (D) Gel showing enrichment of Aha-labeled proteins using the DADPS tag. F – flow-through, W1-5 – washes, E – elution. The band marked by * is monomeric avidin. (E) The structures of the alkyne DADPS tag and the alkyne fragment released upon cleavage.

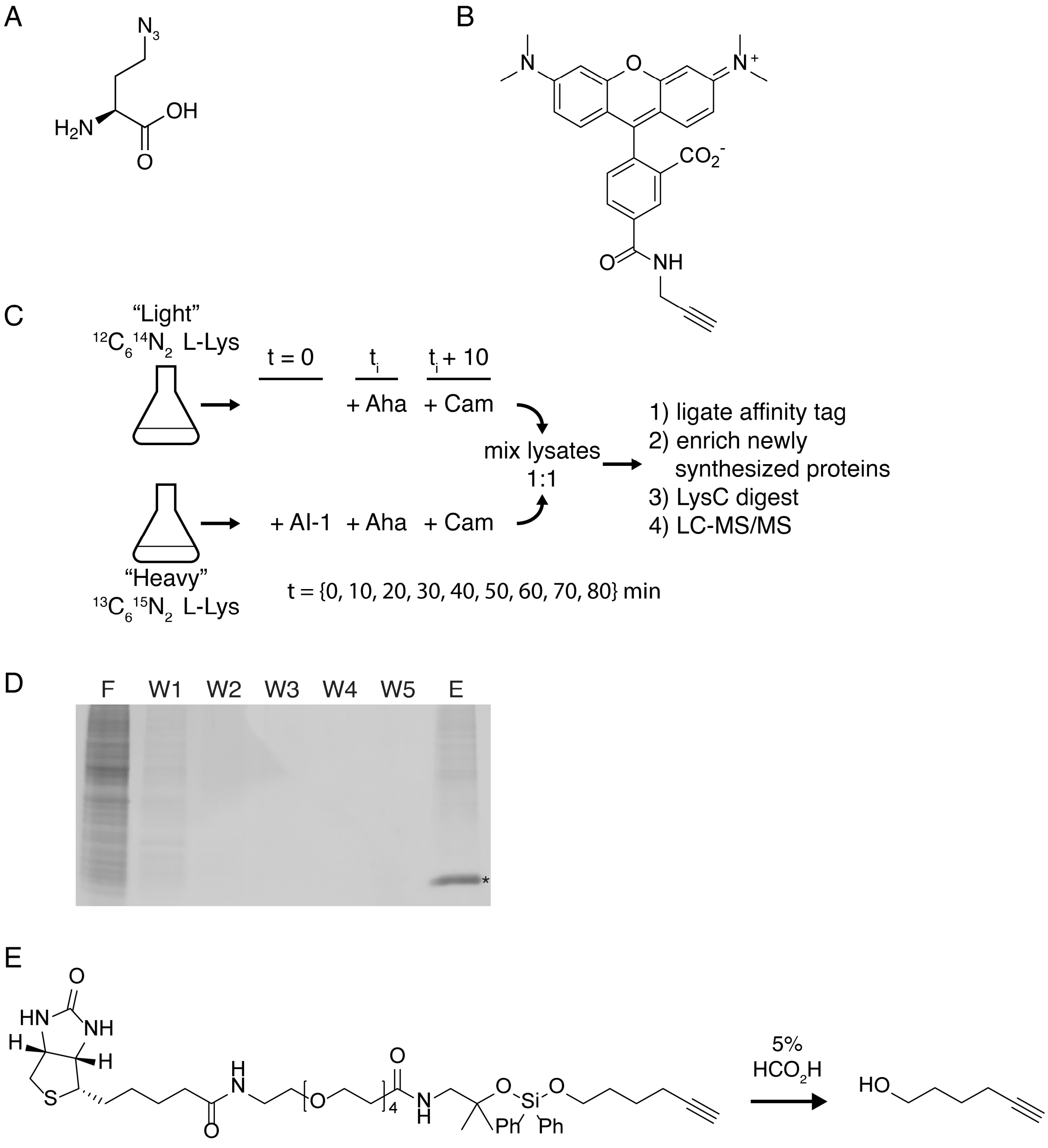

Fig. S2. Confirmation of LuxR peptide (RPRTRLSPLK) quantitation. (A) Masses in the range of 1222.7622 ± 2 ppm; the predicted mass of the RPRTRLSPLK peptide. Orange and blue markers represent normalized ratios of peptides from label swap experiments. (B) An additive model of polypeptide chromatography accurately predicts the retention time of the RPRTRLSPLK peptide. The measured and calculated retention times were 16.02 min and 15.09 min, respectively. (C) Fragmentation spectra of candidate masses in the 1222.7622 ± 2 ppm range were matched to the RPRTRLSPLK peptide by ProteinProspector (v 5.12.4). Red text and lines denote matched fragmentation spectra of the RPRTRLSPLK peptide.

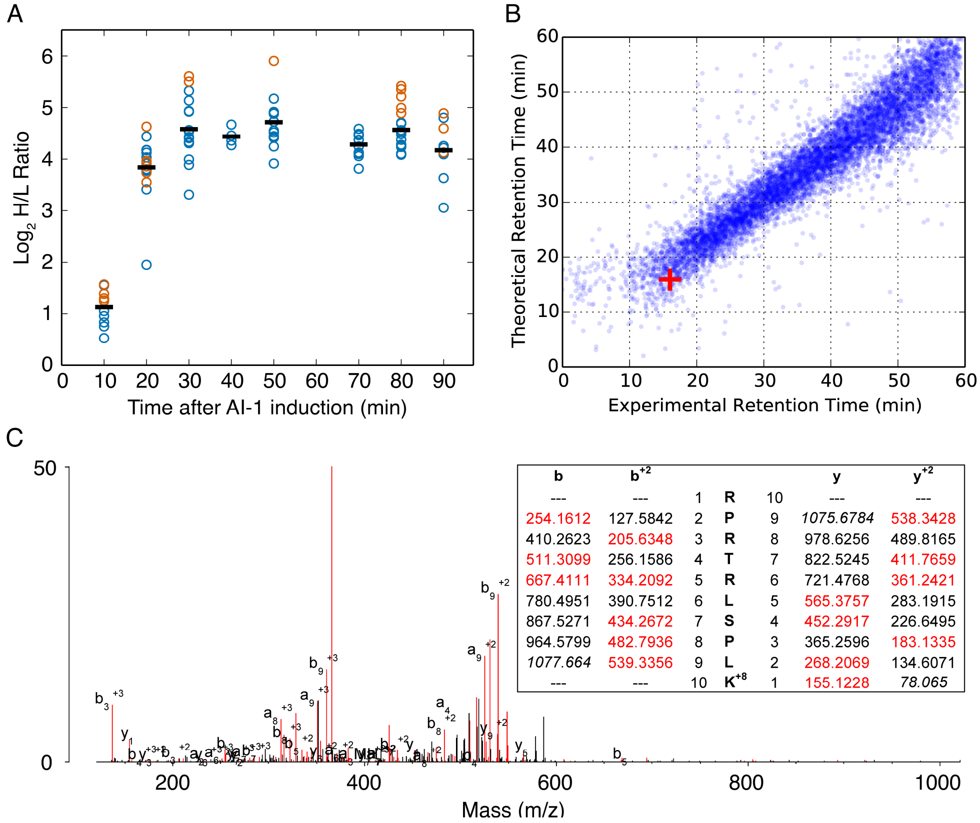


| b | b+2 | | | | y | y+2 |
|---|---|---|---|---|---|---|
| --- | --- | 1 | R | 10 | --- | --- |
| 254.1612 | 127.5842 | 2 | P | 9 | *1075.6784* | 538.3428 |
| 410.2623 | 205.6348 | 3 | R | 8 | 978.6256 | 489.8165 |
| 511.3099 | 256.1586 | 4 | T | 7 | 822.5245 | 411.7659 |
| 667.4111 | 334.2092 | 5 | R | 6 | 721.4768 | 361.2421 |
| 780.4951 | 390.7512 | 6 | L | 5 | 565.3757 | 283.1915 |
| 867.5271 | 434.2672 | 7 | S | 4 | 452.2917 | 226.6495 |
| 964.5799 | 482.7936 | 8 | P | 3 | 365.2596 | 183.1335 |
| *1077.664* | 539.3356 | 9 | L | 2 | 268.2069 | 134.6071 |
| --- | --- | 10 | K+8 | 1 | 155.1228 | *78.065* |

Fig. S3. Summary of measurements from proteomics experiments. (A) Sorted list of MS intensities for all quantified proteins. (B) MS/MS spectra per protein. Number of peptides (C) and quantifications (D) for each protein, calculated separately for each time point.

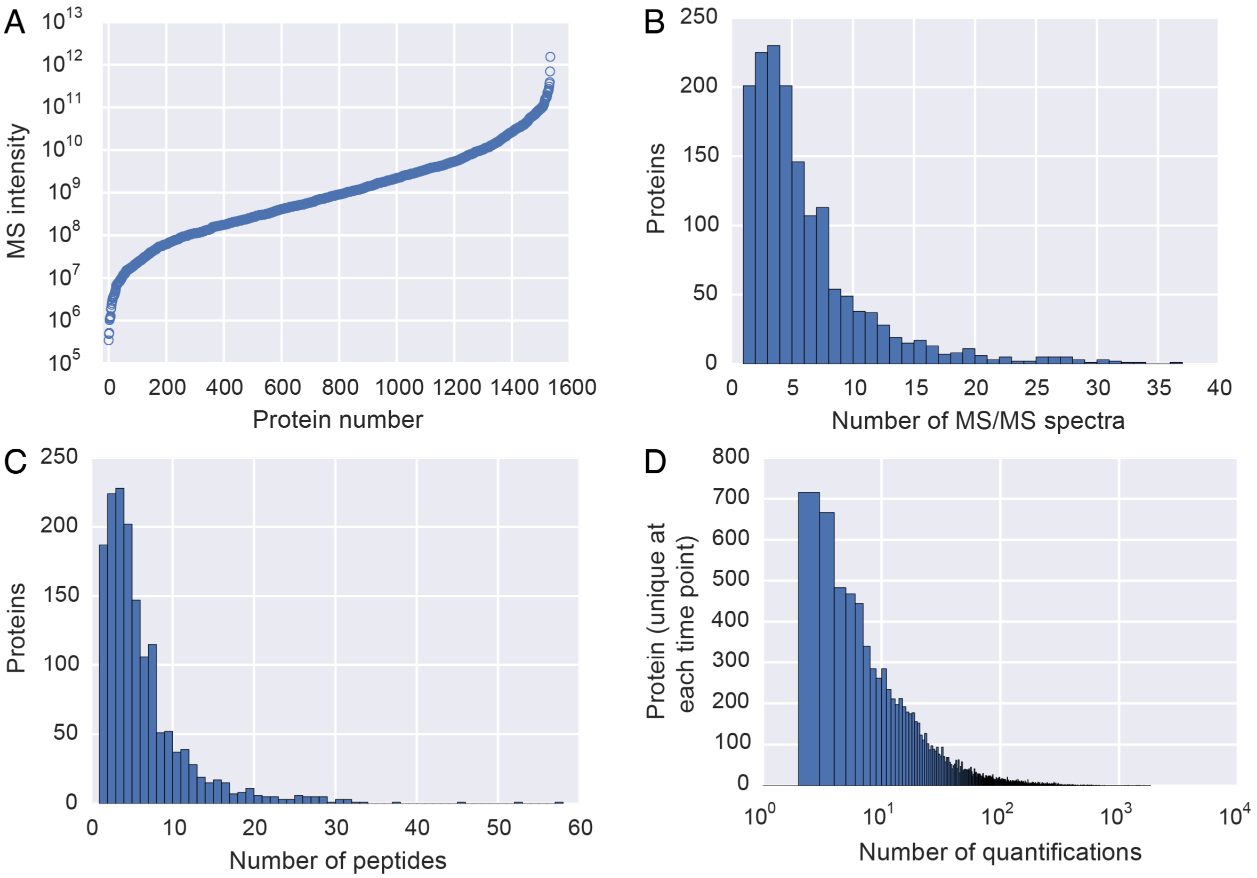

Fig. S4. Comparison of BONCAT proteomics data with the previously measured LuxR, AphA, and quorum-sensing regulons. For each regulon, the subset of genes for which proteins were identified with and without significant regulation is designated.

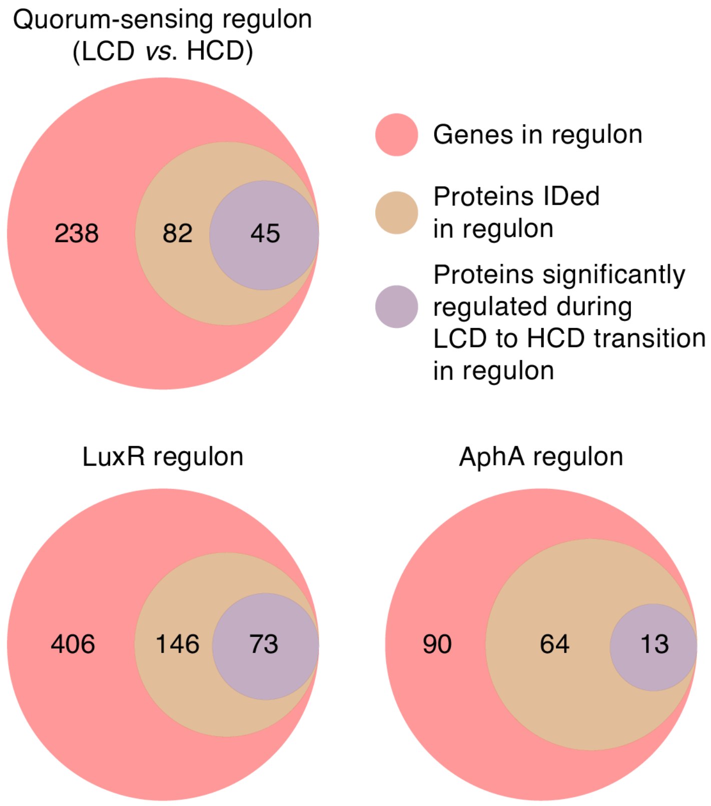

Fig. S5. Gene ontology analysis. Proteins from the significantly regulated gene ontology groups (Fig. 6*B*) are shown on the PCA plot. Groups were scored based on the average distance of proteins from the origin, and groups with fewer than 4 members were excluded. Ontology analysis used a combination of groups from the Gene Ontology (GO) database, and the KEGG Orthology (KO) and KEGG Module (KM) databases.

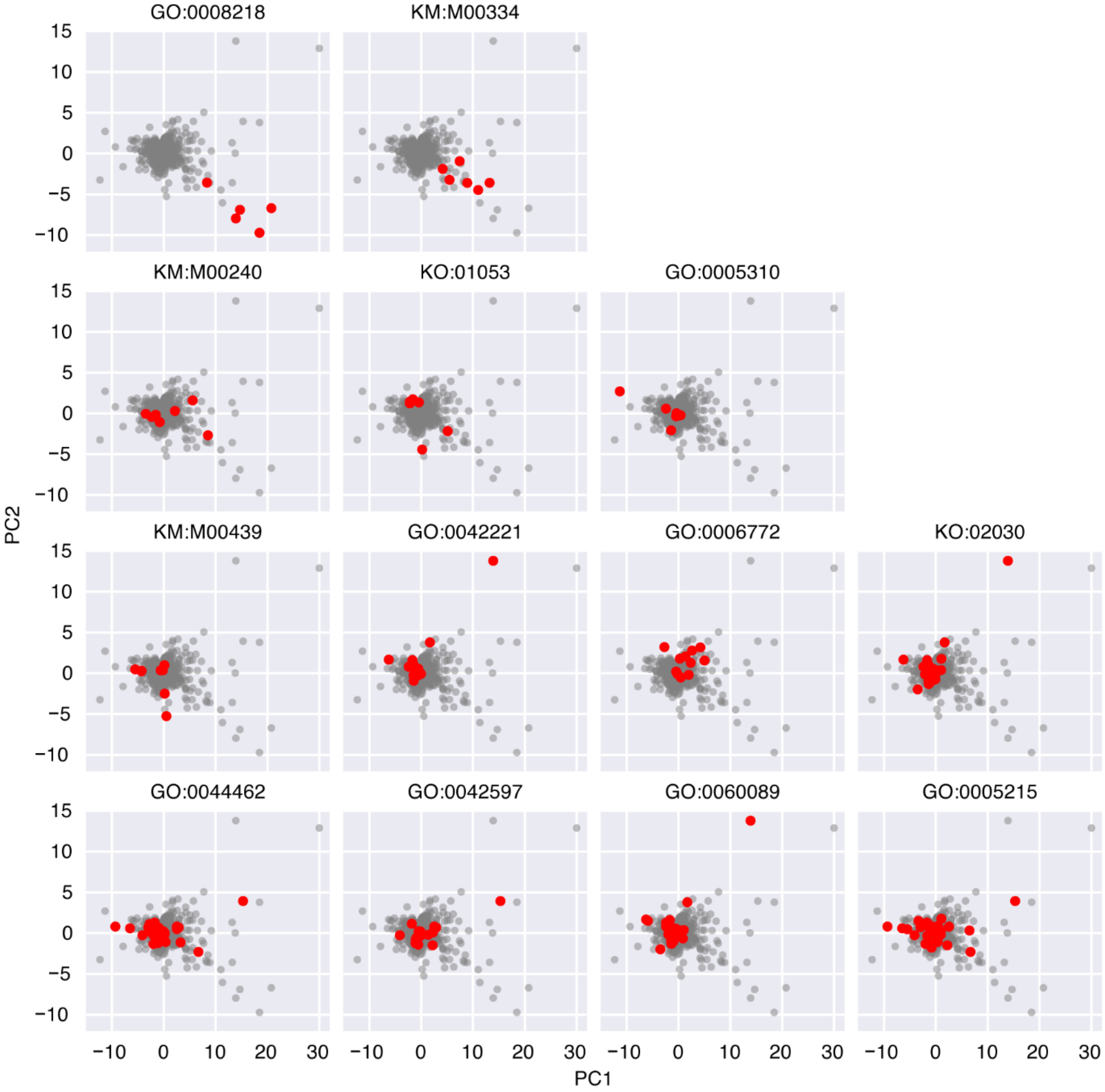


GO:0008218 bioluminescence
KM:M00334 type VI secretion system
KM:M00240 iron complex transport system
KO:01053 biosynthesis of siderophore group nonribosomal peptides
GO:0005310 bicarboxylic acid transmembrane transporter activity
KM:M00439 oligopeptide transport system
GO:0042221 response to chemical
GO:0006772 thiamine metabolic process
KO:02030 bacterial chemotaxis
GO:0044462 external encapsulating structure part
GO:0042597 periplasmic space
GO:0060089 molecular transducer activity
GO:0005215 transporter activity

Fig. S6. (A) The type VI secretion genes in *V. harveyi* are organized into five putative operons. The black asterisk symbol marks the location of the LuxR binding site previously identified by ChIP. Red askterisk symbols mark newly identified LuxR binding sites. (B) Up-regulation of type VI secretion operons at HCD is LuxR-dependent and AphA-independent. Results are from van Kessel et al. (2013). These data show relative gene expression values from Δ*aphA* (JV48), Δ*luxR* (KM669), and Δ*aphA* Δ*luxR* (STR417) *V. harveyi* strains relative to wild-type (BB120; wt) at varying cell densities. (C) EMSAs for reaction mixtures containing 0, 10, 100, or 1000 nM LuxR incubated with 1 nM radiolabeled DNA substrate corresponding to the three TSSS promoter regions for *VIBHAR_05855–56*, *VIBHAR_05865*, or *VIBHAR_05871–72*.

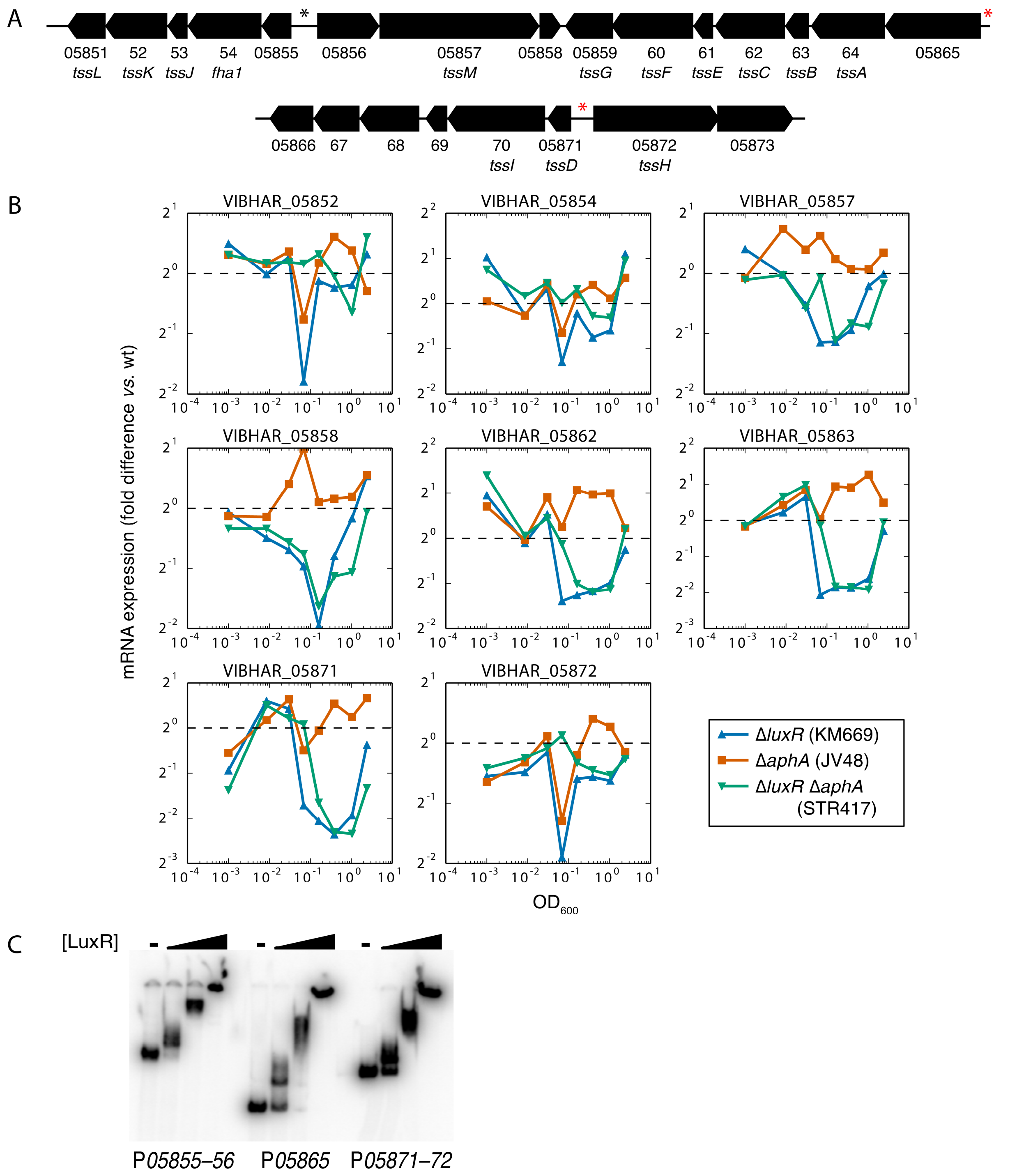

**Supplementary tables**

Table S1. Calculation of Aha incorporation based on total evidence counts, MS-MS counts, and MS intensity provides estimates in a range of 13−17%.

| Measure of abundance | Aha peptides | Met peptides | All peptides | Calculated Aha incorporation |
|---|---|---|---|---|
| Evidence Counts | 26,496 | 131,808 | 158,304 | 16.7% |
| MS-MS Counts | 23,285 | 147,918 | 171,203 | 13.6% |
| MS Intensity | $5.09 \times 10^{11}$ | $2.72 \times 10^{12}$ | $3.23 \times 10^{12}$ | 15.8% |

Table S2. The weights used to transform protein ratios into principal component space. The mean (μ) and standard deviation (σ) of each sample were used to standardize the original variables prior to multivariate analysis.

| | 10 min | 20 min | 30 min | 40 min | 50 min | 60 min | 70 min | 80 min | 90 min | Variance accounted for |
|---|---|---|---|---|---|---|---|---|---|---|
| PC1 | 0.128 | 0.254 | 0.300 | 0.343 | 0.399 | 0.325 | 0.378 | 0.387 | 0.393 | 50% |
| PC2 | 0.583 | 0.407 | 0.428 | 0.196 | -0.018 | -0.360 | -0.259 | -0.220 | -0.168 | 13% |
| PC3 | 0.782 | -0.491 | -0.258 | -0.127 | 0.023 | 0.224 | 0.109 | 0.038 | 0.018 | 9% |
| PC4 | -0.162 | -0.652 | 0.385 | 0.526 | 0.092 | 0.026 | -0.257 | -0.212 | 0.064 | 7% |
| PC5 | 0.042 | 0.296 | -0.473 | 0.523 | -0.273 | 0.392 | -0.119 | -0.386 | 0.147 | 6% |
| PC6 | -0.020 | 0.088 | 0.446 | -0.335 | -0.181 | 0.736 | -0.202 | -0.091 | -0.239 | 5% |
| PC7 | 0.029 | -0.080 | 0.294 | -0.100 | -0.684 | -0.135 | 0.406 | -0.152 | 0.472 | 4% |
| PC8 | -0.031 | -0.047 | 0.042 | 0.321 | -0.175 | 0.018 | 0.593 | 0.032 | -0.713 | 3% |
| PC9 | 0.045 | -0.022 | -0.039 | 0.228 | -0.475 | -0.015 | -0.375 | 0.757 | -0.061 | 3% |
| μ | 0.001 | -0.004 | 0.028 | 0.003 | 0.050 | 0.025 | 0.052 | 0.047 | 0.021 | - |
| σ | 0.203 | 0.250 | 0.311 | 0.259 | 0.451 | 0.381 | 0.444 | 0.575 | 0.375 | - |

Table S3. Timings of significantly regulated proteins that are directly regulated by LuxR and the Qrr sRNAs.

| LuxR-regulated genes | | | Qrr sRNA-regulated genes | | |
|---|---|---|---|---|---|
| Gene | Time first significant (min) | $Log_2$ H/L ratio | Gene | Time first significant (min) | $Log_2$ H/L ratio |
| VIBHAR_06238 | 40–50 | 3.11 | VIBHAR_00417 | 0–10 | 0.66 |
| VIBHAR_06244 | 40–50 | 3.24 | VIBHAR_06667 | 10–20 | 0.78 |
| VIBHAR_02988 | 70–80 | -0.73 | VIBHAR_06666 | 10–20 | 0.92 |
| VIBHAR_06253 | 70–80 | -1.07 | VIBHAR_02446 | 10–20 | 0.75 |
| VIBHAR_01749 | 70–80 | 0.97 | VIBHAR_03459 | 0–10 | 1.13 |
| VIBHAR_01762 | 20–30 | -0.69 | VIBHAR_02959 | 0–10 | 0.74 |
| VIBHAR_00081 | 50–60 | -0.85 | VIBHAR_00046 | 10–20 | -1.22 |
| VIBHAR_03197 | 40–50 | -0.82 | | | |
| VIBHAR_06838 | 50–60 | -3.13 | | | |
| VIBHAR_05086 | 40–50 | 0.96 | | | |
| VIBHAR_02986 | 30–40 | -0.64 | | | |
| VIBHAR_04809 | 0–10 | 0.60 | | | |
| VIBHAR_02041 | 70–80 | 1.08 | | | |
| VIBHAR_06007 | 40–50 | -0.94 | | | |
| VIBHAR_01133 | 20–30 | -0.77 | | | |
| VIBHAR_02617 | 20–30 | -1.00 | | | |
| VIBHAR_06860 | 70–80 | 1.54 | | | |
| VIBHAR_01256 | 50–60 | -1.03 | | | |
| VIBHAR_03248 | 70–80 | -1.63 | | | |
| VIBHAR_05968 | 80–90 | -0.73 | | | |
| VIBHAR_01398 | 70–80 | -0.64 | | | |

Table S4. Oligonucleotides used in this study.

| Name | Sequence |
|---|---|
| P05855-56 | GGGCGAAAGATATCAAGTCTCTCTT |
| P05855-56 | ATTTTCCAATTCCAACTGATTATATGAAGG |
| P05865 | GTTGCTCTTCACTAGCGCTCTTG |
| P05865 | CCTTGTTTCAAGGCTGGTATTTAAA |
| P05871-22 | ATATGCTGGAGTTGGCATCGTTATT |
| P05871-22 | TTTATTCTTTAGAGGAAAAGAGGTGGTC |
| aphA qRT-PCR | ATCCATCAACTCTAGGTGATAAAC |
| aphA qRT-PCR | CGTCGCGAGTGCTAAGTACA |
| luxO qRT-PCR | GCATTCCTGATCTTATTCTGCTCG |
| luxO qRT-PCR | TCCATCCCCGTCATATCAGGTA |
| luxR qRT-PCR | GCAAAGAGACCTCGTACTAGG |
| luxR qRT-PCR | GCGACGAGCAAACACTTC |
| 02788 qRT-PCR | TGTTTAACAGTATACGTACTCGAATCG |
| 02788 qRT-PCR | TCAGTAAATGCATCGGTAGTCAT |
| 05853 qRT-PCR | CAACAGGCATTACGCCAG |
| 05853 qRT-PCR | CGCAAAATAACTGGAGAGGATTG |
| 05857 qRT-PCR | CGATTTTGGTTTCTACCATAGTGG |
| 05857 qRT-PCR | CAATCCATAGATATAGCTTATCTGCATCT |
| 05861 qRT-PCR | GTTATCGTCTTTTAGAGCGGATTG |
| 05861 qRT-PCR | AGGTGAGAATGAATGGATTCGAT |
| 05864 qRT-PCR | GATTGATATCGAACGTTTGCTTACG |
| 05864 qRT-PCR | CTTCACTTCGGATTCCATCATTTC |
| 05871 qRT-PCR | GTGAAACTCAAGGTCACATCAC |
| 05871 qRT-PCR | AGTTCTTGAACTAGGAACTCATCAA |
| 05872 qRT-PCR | GTCTCTGAAAAAGCAAACGAAGT |
| 05872 qRT-PCR | GATTGTCTTAATAAGTTCTCAGAACAATCA |
